# Serous Cystadenocarcinoma Arising in Presumed Vitelline Duct Remnant: A Case Report and Implications in the Management of Cancer of Unknown Primary

**DOI:** 10.1155/2016/4365217

**Published:** 2016-11-24

**Authors:** Li Lei, Jeremy K. Deisch

**Affiliations:** Department of Pathology and Human Anatomy, Loma Linda University Medical Center, Loma Linda, CA, USA

## Abstract

*Background*. Malignant neoplasms arising in Meckel's diverticulum, a vitelline duct remnant, are rare yet well-documented.* Case Presentation*. A 53-year-old previously healthy female presented with an enlarging midline abdominal wall mass. A computed tomography scan revealed a mass involving the linea alba, bilateral rectus abdominis, and subcutaneous fat. Extensive clinical workup failed to demonstrate other lesions, except local and paratracheal/hilar lymphadenopathy. Histopathologic examination of the resected tumor demonstrated a spectrum of serous neoplasia including serous cystadenoma, papillary serous carcinoma with numerous Psammoma bodies, and a poorly differentiated component. Immunophenotypically, the tumor cells were strongly positive for CK7, CK19, CA19.9, and MUC1 but negative for other lineage markers, findings suggestive of pancreatobiliary type differentiation. The patient died of the disease one year after the initial presentation despite chemotherapy, radiation, and surgery.* Conclusion*. We present a case of adenocarcinoma arising from the anterior midline abdominal wall, from presumed vitelline duct remnant, with histologic and immunophenotypic features of serous cystadenocarcinoma of pancreatobiliary origin. Though the origin from vitelline duct remnant is difficult to prove in this single case, understanding tumorigenesis of embryonic remnant origin is potentially important to improve the management of cancer of unknown primary.

## 1. Introduction

Cancer of unknown primary (CUP) is a heterogeneous group and accounts for approximately 3–10% of all cancers. Despite advances in imaging technology and chemotherapy, CUP remains a diagnostic challenge and a therapeutic dilemma. Neoplasms arising in Meckel's diverticulum, a vitelline duct remnant, are rare yet well documented. Most examples comprise carcinoid tumors, gastrointestinal stromal tumors, or gastric-type adenocarcinomas [1–5]. Herein we report the first case of serous cystadenocarcinoma arising from midline abdominal wall and discuss the implications of embryonic remnants, in this case vitelline duct remnant, as a potential origin of tumorigenesis on the management of CUP.

## 2. Materials and Methods

The formalin-fixed paraffin-embedded tissue sections were routinely processed and stained with hematoxylin and eosin. Immunohistochemical stains were performed on the paraffin sections using the following commercially available antibodies: CA19.9, calretinin, CDX-2, CK7, CK19, CK20, ER, GCDFP15, HBME, mammaglobin, MUC1, MUC2, p53, p63, S100, SALL4, synaptophysin, thyroglobulin, TTF-1, and WT1.

## 3. Case Presentation

### 3.1. Clinical Course

**Figure 1:**
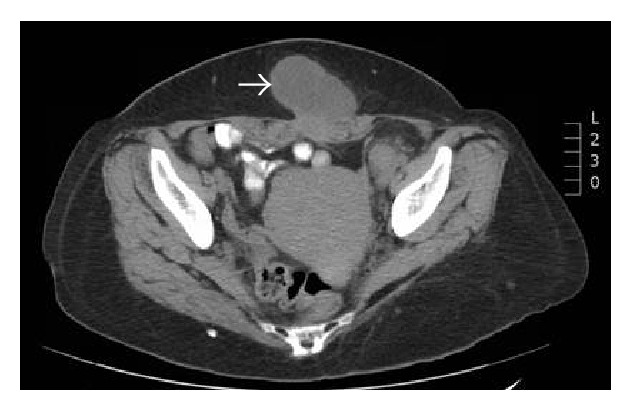
Abdomen and pelvis computed tomography (CT) scan revealed a 6.0-centimeter mass (arrow) involving linea alba, bilateral rectus abdominis, and subcutaneous fat. The mass was not attached to any organs. Also noted was prominent left external iliac and inguinal lymphadenopathy.

**Figure 2:**
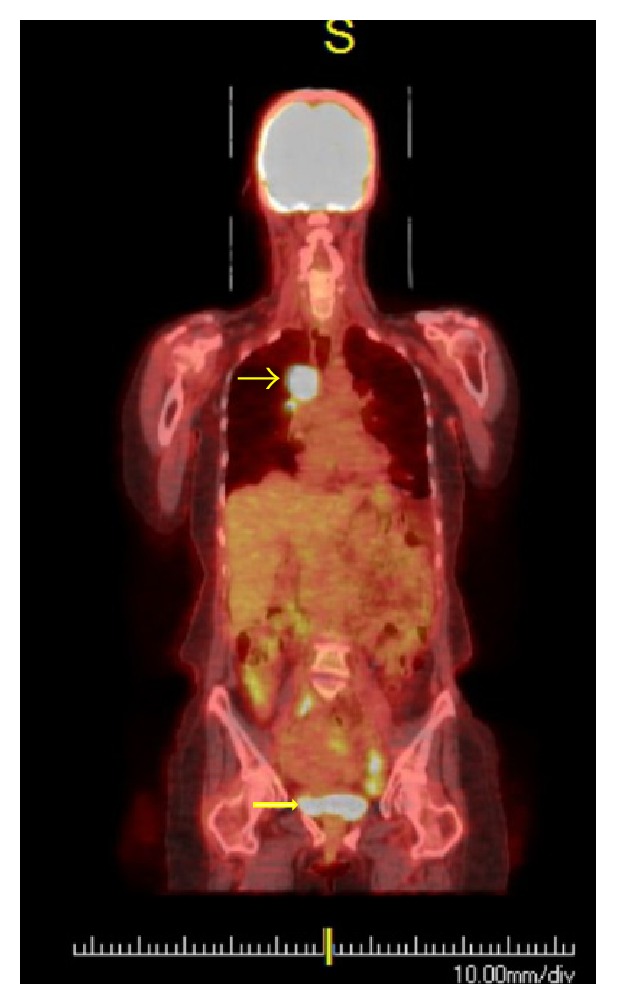
Whole body positron emission tomography-computed tomography (PET-CT) scan highlighted the abdominal wall mass (thick arrow) as well as left inguinal, paratracheal, and hilar (thin arrow) lymphadenopathy. However, no other lesions were identified.

The patient was a 53-year-old previously healthy female who presented with an enlarging midline abdominal wall mass over the past year. She had no family history of cancer. On physical examination, the mass was suprapubic in location, 6.0 × 4.5 cm in size, and nontender. The overlying skin appeared normal. Abdomen and pelvis computed tomography (CT) scan revealed a 6.0-centimeter mass involving linea alba, bilateral rectus abdominis, and subcutaneous fat. There was also prominent left external iliac and inguinal lymphadenopathy (Figure 1); the scan was otherwise unremarkable. A whole body positron emission tomography-computed tomography (PET-CT) scan was performed to evaluate possible sites of origin. The abdominal wall mass and left inguinal lymphadenopathy were highlighted, as was paratracheal and hilar lymphadenopathy (Figure 2).

**Figure 3:**
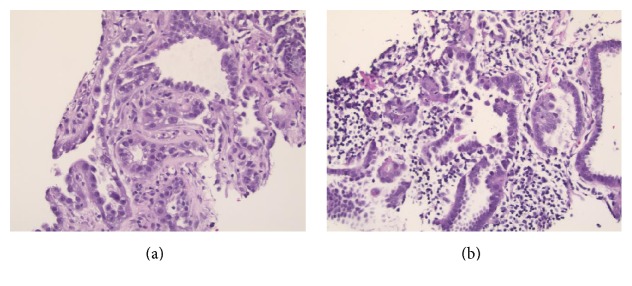
The abdominal wall mass (a) comprised small glands as well as large cystic spaces lined by cuboidal cells with eosinophilic, vacuolated or clear cytoplasm, and variably conspicuous nucleoli. The lymph node (b) showed glandular structures with morphology similar to that seen in the abdominal wall mass and residual lymphoid tissue in the background (hematoxylin-eosin staining, 200x).

Biopsies of the abdominal wall mass (Figure 3(a)) and lymph node (Figure 3(b)) showed small fragments of adenocarcinoma and scant associated lymphoid tissue. An immunohistochemical battery showed the tumor cells to be positive for cytokeratin 7, patchy positive for CA19.9, and patchy CDX2 positive; cytokeratin 20, mesothelial markers, and breast/skin adnexal markers were negative. Although the immunoprofile was not definitive for site of origin, pancreatobiliary origin was suggested. A mediastinal lymph node biopsy showed similar morphology and immunophenotype, consistent with metastatic disease. A serum tumor marker panel showed elevated CA19-9 (65.0 units/mL, reference range <39.9 units/mL), consistent with antigen expression by the tumor cells seen by immunohistochemistry; CA-125 and carcinoembryonic antigen were within reference range.

An extensive workup to determine site of origin was performed, including esophagogastroduodenoscopy, colonoscopy, laparoscopy, endoscopic ultrasound, mammogram, and endometrial biopsy; all studies were within normal limits. A pelvic ultrasound revealed fibroids, but the endometrium and bilateral ovaries were not well-visualized due to the large abdominal wall mass.

**Figure 4:**
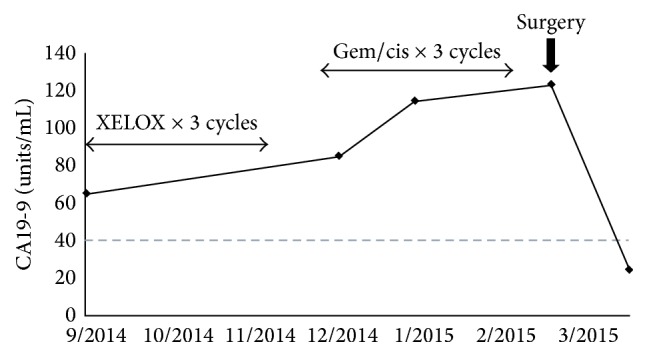
The serum CA19-9 level kept rising in spite of three cycles of XELOX and three cycles of gemcitabine/cisplatin and returned to the reference range following surgical resection of the abdominal wall mass.

The patient underwent chemotherapy for carcinoma of presumed pancreatobiliary origin; the regimen comprised three cycles of XELOX and three cycles of gemcitabine/cisplatin. The mass continued to grow despite chemotherapy, and the serum CA19-9 level kept rising (Figure 4). Eight months after the initial presentation, the patient underwent resection of the abdominal wall mass and left iliac lymph nodes, as well as concurrent hysterectomy and bilateral salpingo-oophorectomy. Intraoperatively, tumor was found to involve the fat, fascia, muscles, and peritoneum of left lower quadrant of abdominal wall and was attached to the pelvic ramus; the bladder dome and bowel were uninvolved. Pathologic examination of uterus and adnexa confirmed uterine leiomyomata, with no evidence of dysplasia, in situ neoplasia, or carcinoma. The resected abdominal wall mass measured 13 cm in size. The cut surface was white and spongy, with areas of white friable tissue. The histologic findings are detailed in the next section. The surgical margins were uninvolved. The serum CA19-9 level normalized following surgery (Figure 4). The mediastinal lymphadenopathy was treated with local radiation.

Four weeks after the surgery, the patient presented with dizziness. Brain magnetic resonance imaging (MRI) showed multiple heterogeneously enhancing lesions, suggestive of metastatic disease. A repeat abdomen and pelvic CT scan showed new mesenteric lymphadenopathy. However, the entire pancreatobiliary system and all other abdominal visceral organs were normal. Neurosurgery was consulted for possible palliative treatment of the brain lesions; the metastatic foci were deemed inoperable. Whole brain radiation was offered. The patient died of disease one year after the initial presentation. Her family declined autopsy.

### 3.2. Histopathologic Examination

**Figure 5:**
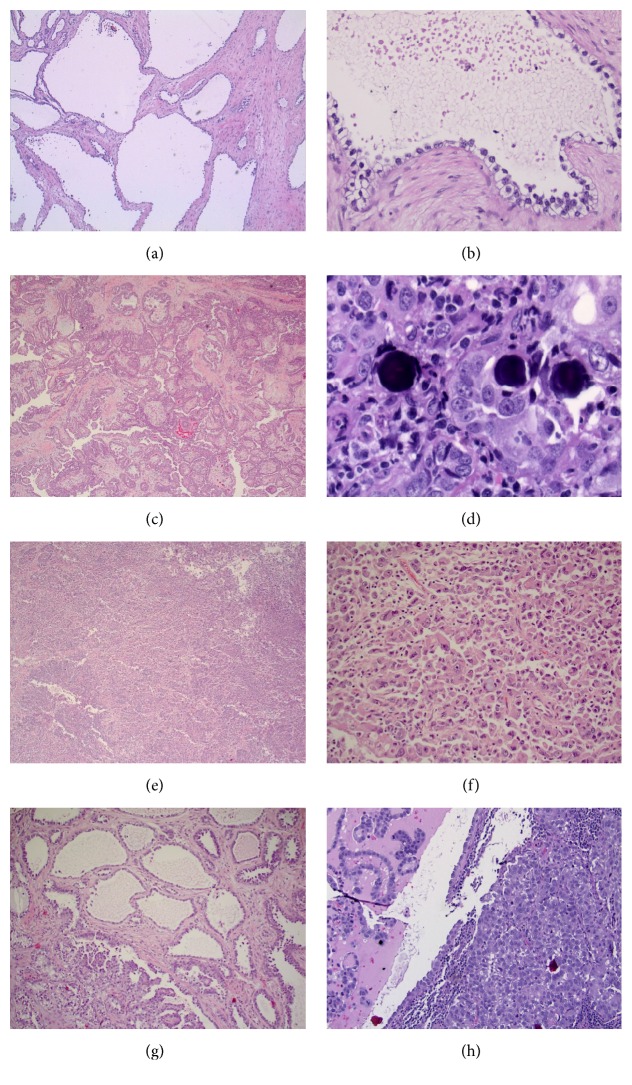
The tumor demonstrated a spectrum of serous neoplasia, with areas of serous cystadenoma representing the better differentiated component (a, b), papillary serous carcinoma with numerous Psammoma bodies representing the areas of high grade tumor (c, d), and solid sheets of poorly differentiated tumor cells representing the dedifferentiated component (e, f). These morphologic features were those of serous neoplasia with progressive dedifferentiation. Transition zones were present (g, h) (hematoxylin-eosin staining; (a), (c), (e), and (g) 40x; (b) 400x; (d) 600x; (f) 200x; (h) 100x).

Sections from the spongy areas of the anterior abdominal wall tumor (Figures 5(a)-5(b)) showed small glands as well as variably sized cystic spaces lined by relatively monotonous cuboidal cells with clear cytoplasm and variably conspicuous nucleoli. In areas, the cystic spaces were lined by mildly atypical cells that protruded into the lumen in a hobnail fashion. In the sections from the spongy areas, complex intracystic papillation was lacking. A mucicarmine stain demonstrated focal intraluminal mucin within the cystic component; there was no intracytoplasmic mucin seen. By PAS staining, the epithelial elements with cytoplasmic clearing showed variable positivity, with decreased staining following diastase pretreatment, findings consistent with cytoplasmic glycogen accumulation.

Sections from the solid areas of the tumor (Figures 5(c)-5(d)) showed progressive decline in the size of the cystic spaces, and the epithelium became increasingly complex. Areas of atypical cells presenting as tubulopapillary and micropapillary structures were encountered. Some papillary structures within the spaces showed complex branching. In the more solid areas, the tumor cells did not form any structures. Instead, they were arranged in large clusters with a background of lymphoplasmacytic inflammation. Laminated spherical calcifications, typical of Psammoma bodies, emerged within the tumor nests. Cytologic atypia showed a progressive increase that correlated with the increasing architectural complexity, becoming pronounced in areas.

In the most undifferentiated areas, the tumor was composed of sheets of discohesive cells with marked cellular atypia and prominent nuclear pleomorphism (Figures 5(e)-5(f)). These cells showed abundant eosinophilic cytoplasm, eccentric enlarged irregular nuclei, and prominent nucleoli. Multinucleation and mitotic figures were frequently encountered. Despite dedifferentiation, cytoplasmic mucin was encountered.

No heterotopic normal tissue was identified in the resected mass. No definite vascular invasion was noted in the sections examined. However, all the histologic components of the large mass were capable of metastasis, as all components were present at lymph nodal metastatic foci; different components in juxtaposition were noted within the same lymph nodes (Figures 5(g)-5(h)).

In summary, histopathologic examination of the tumor demonstrated a spectrum of serous neoplasia, with areas of serous cystadenoma representing the better differentiated component (Figures 5(a)-5(b)), papillary serous carcinoma with numerous Psammoma bodies representing the areas of high grade tumor (Figures 5(c)-5(d)), and solid sheets of poorly differentiated tumor cells representing the dedifferentiated component (Figures 5(e)-5(f)). These morphologic features were those of serous neoplasia with progressive dedifferentiation. Transition zones were present (Figures 5(g)-5(h)).

### 3.3. Immunohistochemistry

**Figure 6:**
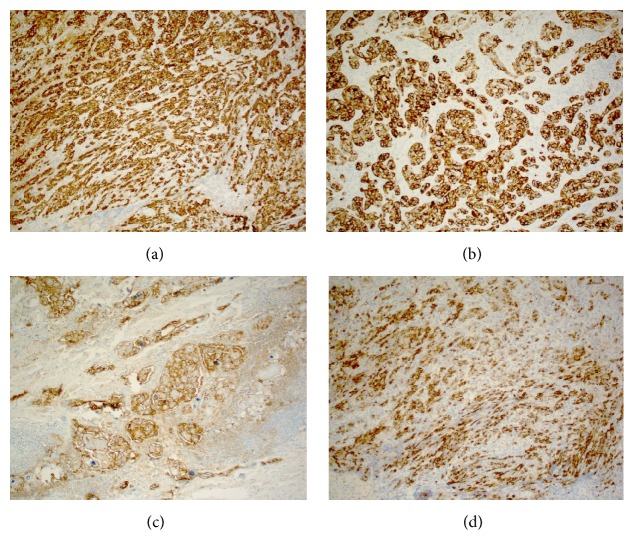
The tumor cells were strongly positive for CK7 (a), CK19 (b), CA19.9 (c), and MUC1 (d) (immunohistochemical staining, 100x).

Immunophenotypically, the tumor cells were strongly positive for CK7, CK19, CA19.9, and MUC1 (Figures 6(a)–6(d)) but were largely negative for CK20, MUC2, TTF-1, thyroglobulin, HBME, GCDFP15, mammaglobin, ER, WT1, calretinin, S-100, synaptophysin, p63, CEA, p53, SALL4, inhibin, and NSE. CDX-2 highlighted occasional tumor cells. While not definitive for a specific site of origin, the immunoprofile strongly suggested pancreatobiliary type differentiation.

## 4. Discussion

This case is unique and diagnostically challenging; the morphologic features were those of serous neoplasm showing progressive dedifferentiation, with immunophenotypical features suggestive of pancreatobiliary differentiation. Serous cystic neoplasms of pancreas are usually benign with negligible malignant potential. When malignant transformation does occasionally develop, serous cystadenocarcinoma of pancreas is typically associated with excellent prognosis even in the presence of metastasis [6, 7]. The present case is unusual in that, despite an extensive workup for an alternative site, lesions outside of the anterior abdominal wall mass and lymph nodal metastases were not encountered; the pancreas and biliary tree were notably normal. In addition, the prominent resistance to pancreatobiliary-targeted chemotherapies and very aggressive biologic behavior were unusual. Moreover, the histological continuity seen in this case suggests the mechanism of progressive dedifferentiation, which has been recognized in many tumor types but is rare for serous neoplasms. In ovary, low grade and high grade serous carcinomas generally represent two distinct tumor pathways driven by mutually exclusive mechanisms. However, at least 16 cases of high grade serous carcinomas of ovaries arising from lower grade lesions have been reported [8–11]. The present case shows similar features, albeit in an example arising outside of the gynecologic tract.

The origin of this unusual tumor is elusive, given the negative pancreatobiliary findings on serial multimodality imaging and the normalization of serum CA19-9 following surgical resection of the tumor; the findings argue against the presence of occult primary and support the abdominal wall mass as the primary site. The remote possibility of diminutive occult primary was considered but would be difficult to confirm even if the autopsy had been performed. From 1944 to 2000, there were 12 publications comprising a total of 884 autopsies on CUP patients. The primary site remained unidentified after autopsy in 27% of cases [12]. This percentage is likely to be higher nowadays given the marked improvement in imaging techniques over the past decades. Nevertheless, the data support the existence of a subset of CUP that cannot be explained by occult or diminutive primary. In these cases, tumors arising in heterotopic tissues or embryonic remnants should be considered [1–5, 13–16].

In regard to the present case, the tumor was most likely of vitelline duct origin, given its anterior midline abdominal wall localization, one site in which vitelline duct remnants may be seen. The presence of heterotopic pancreatic tissues within vitelline duct remnants is well documented. Failure to identify any residual heterotopic pancreatic tissue in this case is not surprising, given the large tumor size and aggressive biologic behavior.

The vitelline duct, also known as omphalomesenteric duct, is a tubular structure that joins the yolk sac to the midgut lumen of the developing fetus. The duct is typically fully obliterated during the 9th week of gestation. In approximately two percent of the population, the duct fails to close. When attached to distal small bowel, it is called “Meckel's diverticulum”; heterotopic tissues such as pancreatic-type and gastric-type are often encountered. Various neoplasms can occur in Meckel's diverticulum, including gastrointestinal stromal tumor, neuroendocrine tumor, and gastric and intestinal adenocarcinoma [1–5]. Cates et al. described an intraductal papillary mucinous adenoma that arose from pancreatic heterotopia within Meckel's diverticulum [5]. Han et al. and Lee et al. reported separately a CA19-9 secreting adenocarcinoma of Meckel's diverticulum without further immunophenotypic characterization [3, 17]. Koh et al. reported a case of CK7 and CA19-9 positive pancreatic-type adenocarcinoma arising in Meckel's diverticulum [4].

Though vitelline duct remnant is most commonly encountered in the form of Meckel's diverticulum in pediatric population, various other anomalies exist and can be encountered in adults, such as persistent omphalomesenteric duct [18] and omphalomesenteric cyst [19–22]. Aydoğan et al. reported a case of omphalomesenteric cyst abscess presented as a suprapubic mass in a 49-year-old female [21]. Sawada et al. described a CA19-9 secreting cystic mass of omphalomesenteric cyst in a 29-year-old male [22]. Our case may represent the first serous cystadenocarcinoma arising in vitelline duct remnant. Though the hypothesis needs further confirmation, the concept may be extrapolated to various CUP presenting as midline abdominal wall masses.

Taking one step further, the possibility of embryonic remnant origin should always be considered in CUP workup. The “embryonic rest hypothesis of cancer origin” was first proposed in the early 19th century and endorsed by many pathologists including Dr. Rudolf Virchow [23]. However, the hypothesis was difficult to prove conclusively (as evidenced by this case) and gradually fell out of favor. There are many embryonic remnants in human body, including the urachus, mesonephric remnant, Müllerian remnant, notochordal remnant, Rathke's pouch remnant, thyroglossal duct remnant, and branchial cleft anomaly. Recently, substantial evidence has accumulated showing that cancer can arise from these embryonic remnants [13–15].

It is important to consider the carcinogenic potential of embryonic remnants when encountering malignancy in unusual or unexpected locations. Diagnostically, although the diagnosis of carcinoma arising within an embryonic remnant requires exclusion of other sites of origin, understanding the potential for embryonic remnant origin may help in limiting confusion when approaching a carcinoma of unknown origin. Therapeutically, carcinomas arising from embryonic rests, despite morphologic and immunophenotypic features similar to those of specific primary sites, may demonstrate different biological behaviors such as escalated aggressiveness and resistance to site-directed chemotherapy, as seen in the present case. Psychologically, it is more difficult for patients as well as their family members to deal with the uncertainty if the origin of carcinoma cannot be identified [24].

CUP management is challenging due to inadequate understanding of underlying tumorigenesis. The role of surgery is uncertain and varies greatly from case to case. In the present case, the development of brain metastases several weeks after the surgery might be coincidental. However, it has been reported that, under certain circumstances, surgical intervention might facilitate tumor dissemination and was associated with increased brain metastasis [25]. In CUP management, molecular genetic profiling to identify the possible site of origin and next-generation sequencing to identify driver mutation and/or actionable molecular targets have been attempted with some success [26, 27]. Molecular tumor profiling predicted tissue of origin in 98% of CUP and may be helpful particularly when immunohistochemical stains are inconclusive [28]. Ross et al. reported that 96% of CUP harbored at least one genomic alteration and 85% of CUP carried one or more potentially targetable genomic alterations [29]. Preliminary results with molecular-targeted agents appear promising and warrant further investigation [30–32]. In retrospect, further molecular testing may have identified a target for directed chemotherapy, which may have prolonged survival in our patient.

## 5. Conclusions

We present a case of adenocarcinoma arising from the anterior midline abdominal wall, from presumed vitelline duct remnant, with histologic and immunophenotypic features of serous cystadenocarcinoma of pancreatobiliary origin. The hypothesis is worth awareness and further testing, as understanding tumorigenesis of embryonic remnant origin can potentially make a difference in the management of cancer of unknown primary.

## Abbreviations

CT: Computed tomography
CUP: Cancer of unknown primary
MRI: Magnetic resonance imaging
PET-CT: Positron emission tomography-computed tomography.
